# SULT2A1 Gene Copy Number Variation is Associated with Urinary Excretion Rate of Steroid Sulfates

**DOI:** 10.3389/fendo.2013.00088

**Published:** 2013-07-12

**Authors:** Jenny Schulze, Maria Johansson, John-Olof Thörngren, Mats Garle, Anders Rane, Lena Ekström

**Affiliations:** ^1^Laboratory Medicine, Division Clinical Pharmacology, Karolinska Institutet, Stockholm, Sweden; ^2^Doping Control Laboratory, Karolinska University Hospital, Stockholm, Sweden

**Keywords:** SULT2A1, SULT1A1, copy number variation, DHEAS, androgens, testosterone

## Abstract

Human cytosolic sulfotransferases (SULT) 2A1 is the main enzyme involved in the sulfate conjugation of dehydroepiandrosterone, a weak androgen, and the main androgen precursor, whereas estrogens are mainly conjugated by SULT1A1. Here we have identified a copy number variation (CNV) polymorphism in the SULT2A1 gene in a Swedish population including healthy men (*N* = 30). Moreover, the CNV of SULT1A1 and SULT2A1 was further characterized in relation to urinary levels of androgen sulfate metabolites before and after an intramuscular dose of 500 mg testosterone enanthate. Individuals expressing two or more CNVs excrete 80 and 40% higher levels of DHEAS (*p* = 0.02) and androsteroneS (*p* = 0.01), respectively as compared to individuals with one gene copy. The mean area under the urine concentration time-curve from time 0 (prior to the administration of 500 mg testosterone) to 15 days post dose values were 80% higher for DHEAS (*p* = 0.046) and testosteroneS (*p* = 0.019) in individuals with two and three SULT2A1 gene copies as compared to individuals with one gene copy. The SULT1A1 CNV on the other hand did not affect the sulfation activity toward the androgens. In conclusion our results indicate that functional CNV polymorphisms in SULT2A1 and SULT1A1 are common in a Swedish population and that SULT2A1 CNV is associated with the urinary concentrations of androgen sulfate metabolites.

## Introduction

Human cytosolic sulfotransferases (SULT) enzymes catalyze the sulfate conjugations of many xenobiotics and endogenous compounds such as hormones. These reactions result in increased water solubility and enhanced urinary excretion of the conjugates. To date, 12 human SULT genes have been identified. Among these SULT1A1 (also known as P-PST) is an enzyme that has been well studied in relation to hormone related disease and detoxification of polycyclic aromatic hydrocarbons (1, 2). Another SULT member SULT2A1 (also known as DHEA ST) is highly expressed in human liver and catalyzes the sulfation of dehydroepiandrosterone (DHEA), the major steroid precursor in humans (3, 4). DHEA itself is a weak androgen which is converted into more active androgens in peripheral tissues (5, 6).

Large inter-individual variations in circulatory levels of DHEAS have been observed. Three SULT2A1 single nucleotide polymorphisms altering the amino acid sequence have been identified and associated with altered SULT2A1 activity and DHEA:DHEAS ratio in the circulation (7). Circulatory DHEAS is considered to represent the androgen pool and high concentration of DHEAS has been associated with diseases and disorders such, i.e., PCOS, cancer, and obesity (8, 9). In addition, the urinary concentration of DHEAS, as well as other androgen sulfates vary manifold between individuals (10–12). This large inter-individual variation in urinary excretion rate may be due to genetic variation in SULT enzymes; however this has not been studied.

A copy number variation (CNV) polymorphism has been identified in SULT1A1. It was shown that individuals display between one and five SULT1A1 gene copies and the number of gene copies have been associated with the enzyme activity (13). Recently it was found that SULT2A1 may exist in different CNVs (15). However, there are no studies confirming this CNV in a human population. CNV in other phase II hormone conjugating enzymes, i.e., uridine diphosphate-glucuronyltransferases (UGTs) has been well characterized (14). We have previously shown that a large extent of the variation of urinary concentration of androgen glucuronides can be ascribed to genetic variation in (UGTs). A CNV of UGT2B17 is strongly associated with testosterone-glucuronide concentrations in urine, both at baseline (15) and after the administration of a supra-physiological dose of testosterone (16).

Here we aim to investigate if SULT2A1 display variation in copy numbers in a Swedish population, and if there is an association with SULT1A1 and SULT2A1 CNV genotype and the urinary excretion of different androgen sulfates prior to and after the administration of testosterone.

## Materials and Methods

### Subjects and design

Study subjects included 30 healthy male volunteers aged 18–50 years. The study population has been described in more detail elsewhere (16). All participants gave informed consent consistent with the approval of the Ethics Review Board, Karolinska Institutet, Stockholm. The participants were given 500 mg testosterone enanthate as a single intra muscular (i.m) dose of Testoviron Depot (kindly provided by Schering Nordiska AB, Solna, Sweden) equivalent to 360 mg testosterone. Urine samples were collected for analyses before administration (day 0) and on days 1–9, 11, 13, and 15 after dose. All samples were collected between 07:00 and 11:00 h. Adverse drug reactions were monitored during the study period. No major adverse drug reactions were registered. No follow-up was needed. The study was conducted according to the Helsinki Declaration and the ICH Harmonized Tripartite Guideline for Good Clinical Practice.

### Urinary analysis

The concentrations of sulfated androgens were analyzed using a LC-MS/MS method as previously described (10). Briefly, 1 mL and 20 lL ISTD samples were added to SPE Oasis HLB 96-well plates and washed with 0.1% acetic acid, 0.1% ammonia, and 10% MeOH. The analytes were eluted with acetone and after evaporation the residue was reconstituted with 20% MeOH. Waters Acquity ultra performance liquid chromatographic (UPLC) system was used to perform the separation on Waters Acquity UPLC BEH RP18 column 50 9 2.1 mm with 1.7 lm particles. The mobile phases were 5 mM NH4Ac adjusted by ammonia to pH 9.6 (A) and MeOH (B). A Waters Micromass Quattro Premier triple-quadrupole instrument (Waters Associates, Manchester, UK) operating with fast polarity switching in multiple reaction mode (MRM) was used to detect the target analytes.

### SULT1A1 and SULT2A1 gene copy number variation

Genomic DNA was extracted from 200 μL blood using Qiagen Mini Kit. Twenty nanograms genomic DNA was used in each reaction together with 2X TaqMan Universal Master Mix (Applied Biosystems, Foster City, CA, USA). The SULT1A1 and SULT2A1 CNV polymorphisms were analyzed by real-time polymerase chain reaction analysis using premade assays Hs04461762_cn and Hs03013147_cn (Applied Biosystems). Expression of albumin was quantified as an endogenous control as described (17). Both reactions were run in 15 μL reactions. The PCR profile consisted of an initial denaturation step at 95°C for 10 min followed by 40 cycles of 92°C for 15 s and 60°C for 1 min.

### Data evaluation

Integration, calibration, and data evaluation was performed by the TargetLynx software (Waters Associates, Manchester, UK). The between subject variation in urine dilution was corrected for by dividing the concentration values by the urinary creatinine (cr) concentration, which was determined by colorimetric analysis (DRI Creatinine-Detect Test; Thermo Fisher Scientific, Waltham, MA, USA). The areas under the curve (AUC) of the different urinary steroids were calculated using the trapezoidal rule. Statistical analyses were performed by Kruskal Wallis followed by Dunn’s Multiple Comparison Test, since the data were not normally distributed.

## Results

### Gene copy number analysis

The distribution of SULT1A1 copy numbers were; individuals with one gene copy (i.e., one deletion) *N* = 6 (20%), two copies *N* = 16 (53%), and three copies *N* = 8 (26%), respectively. The distribution of SULT2A1 copy numbers were; individuals with one copy *N* = 7 (23%), two copies *N* = 9 (60%), and three gene copies *N* = 4 (13%), respectively.

### SULT1A1 and SULT2A1 CNV and urinary baseline levels of steroid sulfates

**Figure 1:**
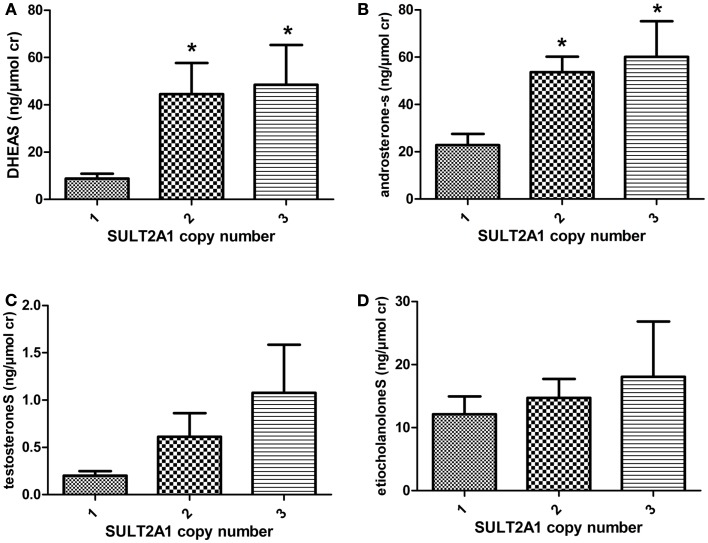
**The mean urinary concentration of (A) DHEAS (B) androsteroneS (C) testosteroneS and (D) etiocholanoloneS in different SULT2A1 genotype panels**. The androgen conjugate concentration was normalized against creatinine (cr) concentration. Individuals with two and three SULT2A copy number variation excrete higher levels of DHEAS and androsteroneS.

There was a significant correlation between urinary concentration of DHEAS (*p* = 0.02) and androsteroneS (*p* = 0.01) and the number of SULT2A1 CNV, Figures 1A,B. Individuals displaying one SULT2A1 gene copy excreted lower levels of DHEAS and androsteroneS (8.8 and 22.9 ng/μmol cr) compared to individuals with two copies (44.5 and 53.6 ng/μmol cr) and three SULT2A1 gene copies (48.5 and 60.1 ng/μmol cr). There were no significant correlation between SUL2A1 CNV and urinary concentration of testosteroneS and etiocholanoloneS, Figures 1C,D. There was no significant association between SULT1A1 CNV and urinary levels of any androgen sulfates investigated.

### SULT2A1 CNV and urinary AUC of steroid sulfates post testosterone administration

**Figure 2:**
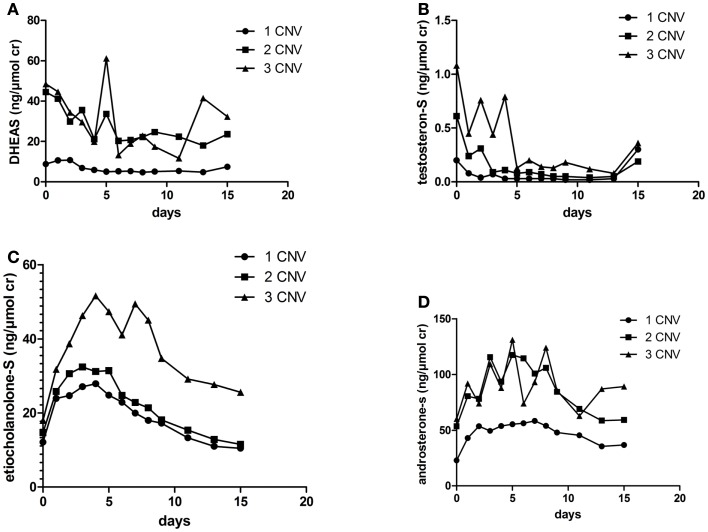
**Urinary (A) DHEAS (B) testosteroneS (C) etiocholanoloneS and (D) androsteroneS excretion (ng/μmol) for 15 days in the different SULT2A1 genotype groups after an i.m injection of 500 mg testosterone enanthate**.

The mean AUC for DHEAS during 15 days post dose were 44.5, 279, and 300 ng/μmol cr in individuals with one, two, and three gene copies, respectively (*p* = 0.046), Figure 2A. The mean AUC for testosteroneS during 15 days were 2.2, 7.4, and 11.6 nmol/μmol cr in individuals with one, two, and three gene copies, respectively (*p* = 0.019), Figure 2B. There were no significant correlations between urinary AUC for etiocholanoloneS, androsteroneS, and SULT2A1 CNV, Figures 2C,D.

There were no significant association between SULT1A1 CNV and urinary AUC of androgen sulfates investigated.

## Discussion

Piper et al. showed that there is a large inter-individual variation (500-fold) in the urinary excretion rate of DHEAS (11). However, there are no studies investigating the inter-individual variability of urinary levels of DHEAS in relation to genotype. We show for the first time that SULT2A1 CNV is functional and that the number of SULT2A1 copies is associated with the baseline concentration of DHEAS, as well as the baseline concentration of the DHEA metabolite androsterone. *In vitro* studies have shown that in addition to DHEA, SULT2A1 also possesses high sulfation activity toward androsterone (4). There was lower sulfation activity in individuals with one SULT2A1 copy compared to individuals with two or more copies for all steroid metabolites investigated except etiocholanoloneS. Our results demonstrate that SULT2A1 exerts activity not only toward DHEA but also toward several DHEA metabolites *in vivo*.

After administration of exogenous testosterone to healthy volunteers, the urinary excretion rate of sulfate metabolites was significantly associated with SULT2A1 CNV. The urinary concentration of DHEAS and testosteroneS decreased approximately 50 and 80% respectively, after the administration of testosterone enanthate. This is probably a result of a feedback mechanism on the hypothalamic-pituitary axis. However, a significant SULT2A1 genotype association with the excretion of both DHEAS and testosteroneS, even at the low concentrations observed post dose further strengthen the finding that DHEA and testosterone are substrates of SULT2A1 *in vivo*. On the other hand the urinary levels of the testosterone metabolites androsteroneS and etiocholanoloneS increased as a result of the administrated testosterone. The association between CNV of SULT2A1 and AUC of androsteroneS was not statistically significant. However as the study sample was small with only seven individuals with one gene copy, the power might have been insufficient to demonstrate any significance. Our AUC results are in agreement with our baseline finding, supporting that these androgen metabolites are sulfated by SULT2A1 *in vivo*.

The allele distribution of SULT1A1 was in agreement with a previous study reporting a 25% frequency of three SULT1A1 copies in Caucasians (13) but opposite to other studies reporting a 5% occurrence of one copy number (13, 18). There was no correlation between CNV of SULT1A1 and the urine concentration of the steroid sulfates. Even though SULT1A1 has been shown to be involved in hormone metabolism, this enzyme has been shown to be more important in the sulfation of estrogens (2).

For ethical and medical reason, testosterone was administered to men only. Sulfation of androgens are important also in women, and genetic variation in different SULTs have been associated with altered risk for PCOS and breast cancer treatment (19, 20) and it would be of interest to study this functional CNV in relation to the metabolism of endogenous and exogenous androgens in females in future studies.

DHEAS is found at high concentrations in the circulation and has in addition to hormone related cancers and PCOS also been shown to be involved in the risk of cardiovascular diseases (21, 22). SULT2A1 expression is down-regulated in hepatocellular carcinoma and correlated with higher grade and stage of cancer (23). Additionally, SULT2A1 has been shown to be involved in the metabolism and activation of carcinogenic compounds and drugs (24, 25). It is possible that SULT2A1 CNV polymorphism may contribute to individual variation in response to drugs that undergo sulfation as well as altered risk for cancer and other diseases.

Variability in CNV is a common phenomenon in the human genome, and has been observed for a number of enzymes involved in biotransformation, i.e., UGT2B17, CYP2D6, and GSTM (26). This is the first time SULT2A1 CNV has been studied in a human population, and our results confirm that this putative polymorphism exists. According to the provider (Applied Biosystems) of the SULT2A1 gene copy assay used in this study the deletion consists of 2849 bp at the 3′-end of the SULT2A1 gene, at chromosome position 19q13.33. Jung et al. identified a 150 000 bp homozygous SULT2A1 gene deletion at position 19q13.32 in a pulmonary inflammatory myofibroblastic tumor (27). It is possible that different CNV exists throughout the SULT2A1 gene. Further studies are warranted in order to characterize the genetic make-up of this CNV and the phenotypic consequences.

## Conflict of Interest Statement

The authors declare that the research was conducted in the absence of any commercial or financial relationships that could be construed as a potential conflict of interest.
